# Gene expression of *Pocillopora damicornis* coral larvae in response to acidification and ocean warming

**DOI:** 10.1186/s12863-024-01211-3

**Published:** 2024-03-08

**Authors:** Youfang Sun, Yi Lan, Nils Rädecker, Huaxia Sheng, Guillermo Diaz-Pulido, Pei-Yuan Qian, Hui Huang

**Affiliations:** 1 Key Laboratory of Tropical Marine Bio-resources and Ecology, Guangdong Provincial Key Laboratory of Applied Marine Biology, South China Sea Institute of Oceanology, Chinese Academy of Sciences, 510301 Guangzhou, China; 2 Department of Ocean Science, The Hong Kong University of Science and Technology, Hong Kong, China; 3 State Key Laboratory of Marine Environmental Sciences, Xiamen University, 361101 Xiamen, China; 4 Laboratory for Biological Geochemistry, School of Architecture, Civil and Environmental Engineering, École Polytechnique Fédérale de Lausanne (EPFL), 1015 Lausanne, Switzerland; 5 CAS-HKUST Sanya Joint Laboratory of Marine Science Research and Key Laboratory of Tropical Marine Biotechnology of Hainan Province, Tropical Marine Biological Research Station in Hainan, Chinese Academy of Sciences, 572000 Sanya, China; 6 Southern Marine Science and Engineering Guangdong Laboratory, 511458 Guangzhou, China; 7 School of Environment and Science, Coastal and Marine Research Centre, and Australian Rivers Institute, Griffith University, Nathan Campus, 4111 Brisbane, Queensland Australia

**Keywords:** Transcriptomics, *Pocillopora damicornis*, Coral larvae, Ocean warming, Acidification

## Abstract

**Objectives:**

The endosymbiosis with Symbiodiniaceae is key to the ecological success of reef-building corals. However, climate change is threatening to destabilize this symbiosis on a global scale. Most studies looking into the response of corals to heat stress and ocean acidification focus on coral colonies. As such, our knowledge of symbiotic interactions and stress response in other stages of the coral lifecycle remains limited. Establishing transcriptomic resources for coral larvae under stress can thus provide a foundation for understanding the genomic basis of symbiosis, and its susceptibility to climate change. Here, we present a gene expression dataset generated from larvae of the coral *Pocillopora damicornis* in response to exposure to acidification and elevated temperature conditions below the bleaching threshold of the symbiosis.

**Data description:**

This dataset is comprised of 16 samples (30 larvae per sample) collected from four treatments (Control, High *p*CO_2_, High Temperature, and Combined *p*CO_2_ and Temperature treatments). Freshly collected larvae were exposed to treatment conditions for five days, providing valuable insights into gene expression in this vulnerable stage of the lifecycle. In combination with previously published datasets, this transcriptomic resource will facilitate the in-depth investigation of the effects of ocean acidification and elevated temperature on coral larvae and its implication for symbiosis.

## Objective

The symbiotic relationship between stony corals and their algal endosymbionts (Symbiodiniaceae) is the cornerstone underlying the productivity of coral reefs in the oligotrophic environment in the tropical ocean [1–4]. Yet, this dependence on symbiotic partners may prove to be the Achilles’ heel of corals in the Anthropocene [5]. Elevated temperature and acidification have the potential to perturb the symbiosis, leading to coral bleaching, mortality, and eventually reef degradation [6–8].

Studies of gene expression have proven valuable for detecting genes and signaling pathways underlying the disturbance of the symbiotic relationship between the coral host and its endosymbionts [9–11]. For example, studies on *Stylophora pistillata* revealed that heat stress alters the energy metabolism of the host, thereby promoting catabolic ammonium release and the destabilization of symbiotic nutrient recycling [12, 13]. However, most of our knowledge of gene expression in corals is based on studies of adult coral colonies [14–17]. In comparison, other stages of the coral lifecycle have received less attention to date.

Coral larvae represent a bottleneck in the reproduction of corals and have been suggested to be susceptible to elevated temperatures and ocean acidification [18, 19]. Larvae of the widespread brooding coral *Pocillopora damicornis* have emerged as powerful model systems to study the effects of future ocean conditions on gene expression of symbiotic larvae [9, 11, 20, 21]. Jiang et al. recently suggested that the bleaching threshold of *P. damicornis* larvae harboring the symbiont *Durusdinium trenchi* was between 32 and 33℃ [21]. Hence, we aimed to improve our understanding of the sub-bleaching stress response of *P. damicornis* larvae by studying the effects of acidification and warming on host and symbiont gene expression. Here, we present a transcriptomic dataset for 16 samples of coral larvae exposed to four treatments combing modulated *p*CO_2_ (450 or 1,000 µatm) and temperature (29 or 32℃) conditions (data file 1 [22]).

## Data description

### Sample collection

Detailed information on the larval collection and experimental setup was previously described in [22]. In brief, *P. damicornis* larvae were collected in Sanya, Hainan Island, China, on September 15, 2018, and pooled together for the subsequent experiments. Coral larvae were exposed to four different treatment conditions for 5 days, i.e., control, high *p*CO_2_, high temperature, combined. The mean temperatures for each treatment were 29.25 ± 0.01 (control, mean ± standard error, 28.90 ± 0.01 (high *p*CO_2_), 32.24 ± 0.01 (high temperature), and 31.86 ± 0.01 °C (combined). The mean *p*CO_2_ for each treatment was 465 ± 18 (control), 1012 ± 42 (high *p*CO_2_), 437 ± 16 (high T), and 966 ± 35 µatm (combined).

### RNA extraction, library construction and transcriptome sequencing

On day five of the experiment, thirty larvae (*n* = 4 replicates per treatment) were preserved in liquid nitrogen for RNA extraction. Total RNA of each larval sample was extracted using a TRIzol® Reagent RNA Isolation Kit (Invitrogen, Grand Island, NY, United States) following the manufacturer’s instructions. The concentration of total RNA was quantified using the Qubit BR RNA Assay Kit (Thermo Scientific), and its integrity was evaluated using an Agilent 2100 Bioanalyzer (Agilent Technologies), according to the manufacturer’s instructions. PolyA + selection and subsequent messenger RNA (mRNA) library preparation were done using the TruSeq Stranded mRNA Library Kit (Illumina), according to the manufacture’s instructions. All libraries were sequenced on the Illumina Hiseq X Ten platform to obtain paired-end reads with a length of 150-bp.

**Table 1:** Overview of data files/data sets

Label	Name of data file/data set	File types (file extension)	Data repository and identifier (DOI or accession number)
Data file 1	Sun_et al_GeneExpressionData	MS Excel file (.xlsx)	Figshare (10.6084/m9.figshare.24474484.v4) [22]
Data file 2	Sun_et al_DetailedMethods	MS Word file (.docx)	Figshare (10.6084/m9.figshare.24474484.v45) [22]
Data set 1	Transcriptomic dataset for larval *P. damicornis*	FASTQ files (.fastq)	NCBI Sequence Read Archive (https://identifiers.org/bioproject:PRJNA976470) [23]

### Gene expression analysis

Sequences were quality trimmed and reads were split and aligned to the gene sequences of *P. damicornis* and *D. trenchii* respectively. This yielded 12–16.5 and 1.9–4.3 million mapped read pairs per sample for the host and Symbiodiniaceae (data file 1 [22]), respectively. Differential gene expression analysis revealed that high *p*CO_2_ alone had no significant effects on host gene expression (*P* < 0.05; Data file 1 [22]). In contrast, high temperature caused pronounced changes in host gene expression with 211 (16 upregulated vs. 195 downregulated) differentially expressed genes compared to control conditions (*P* < 0.05; Data file 1 [22]). Gene ontology (GO) enrichment analyses revealed that these changes in gene expression translated into 62 enriched GO terms under high temperature conditions (*P* < 0.05; Data file 1 [22]), including several processes related to development (e.g., lipoprotein metabolic process, protein autoprocessing, growth hormone receptor signaling pathway, cytolysis, regulation of growth, and negative regulation of cell population pathway). Similarly, combined high *p*CO_2_ and temperature treatment caused differential expression of 439 (274 upregulated vs. 165 downregulated) host genes (*P* < 0.05; Data file 1 [22]). GO enrichment analysis further identified 15 significantly enriched GO terms under combined treatment conditions (*P* < 0.05; Data file 1 [22]), including several processed related to innate immunity (e.g., negative regulation of NF-kappa B transcription factor activity, innate immune response, regulation of apoptotic process). Symbionts showed 8 (5 upregulated vs. 3 downregulated) differentially expressed genes in response to high *p*CO_2_ conditions (*P* < 0.05; Data file 1 [22]). GO enrichment analysis further identified 18 enriched GO terms under high *p*CO_2_ conditions (*P* < 0.05; Data file 1 [22]), including several processes related to catabolism (e.g., cellulose catabolic process, protein catabolic process, polysaccharide catabolic process). High temperature conditions caused differential expression of 40 (18 upregulated vs. 22 downregulated) algal symbiont genes (*P* < 0.05; Data file 1 [22]). GO enrichment analysis further identified 51 enriched GO terms under high temperature conditions (*P* < 0.05; Data file 1 [22]), including several processes related to cellular transport (e.g., proton tranmembrane transport, transmembrane transport, ammonium transmembrane transport). Lastly, combined high *p*CO_2_ and temperature conditions caused differential expression of 14 (8 upregulated vs. 6 downregulated) algal symbiont genes (*P* < 0.05; Data file 1 [22]). GO enrichment analysis identified 28 enriched GO terms under combined treatment conditions (*P* < 0.05; Data file 1 [22]), including several processes related to cellular nitrogen metabolism (e.g., glutamine metabolic process, L-alanine catabolic process, cellular nitrogen compound metabolic process).

## Limitations

*P. damicornis* larvae usually settle around 7 days after their release. As such, the experimental time frame did not permit a gradual acclimation of larvae to the treatment conditions. Identified transcriptomic response may thus include acute stress response arising from a lack of acclimation by the larvae.

## Data Availability

All raw sequencing reads of the coral *P. damicornis* are available in the NCBI Sequence Read Archive under BioProject accession number SRR24750928-SRR11802621 and PRJNA976470 (https://identifiers.org/bioproject:PRJNA976470). In addition, results of differential gene expression and gene ontology enrichment analysis are available via FigShare (10.6084/m9.figshare.24474484.v4). Please see Table 1 and references [22, 23] for details and links to the data.
